# Estimation of Gait Mechanics Based on Simulated and Measured IMU Data Using an Artificial Neural Network

**DOI:** 10.3389/fbioe.2020.00041

**Published:** 2020-02-05

**Authors:** Marion Mundt, Arnd Koeppe, Sina David, Tom Witter, Franz Bamer, Wolfgang Potthast, Bernd Markert

**Affiliations:** ^1^Institute of General Mechanics, RWTH Aachen University, Aachen, Germany; ^2^Institute of Biomechanics and Orthopeadics, German Sport University Cologne, Cologne, Germany

**Keywords:** machine learning, artificial neural networks, wearable sensors, inertial sensors, motion analysis, data simulation

## Abstract

Enhancement of activity is one major topic related to the aging society. Therefore, it is necessary to understand people's motion and identify possible risk factors during activity. Technology can be used to monitor motion patterns during daily life. Especially the use of artificial intelligence combined with wearable sensors can simplify measurement systems and might at some point replace the standard motion capturing using optical measurement technologies. Therefore, this study aims to analyze the estimation of 3D joint angles and joint moments of the lower limbs based on IMU data using a feedforward neural network. The dataset summarizes optical motion capture data of former studies and additional newly collected IMU data. Based on the optical data, the acceleration and angular rate of inertial sensors was simulated. The data was augmented by simulating different sensor positions and orientations. In this study, gait analysis was undertaken with 30 participants using a conventional motion capture set-up based on an optoelectronic system and force plates in parallel with a custom IMU system consisting of five sensors. A mean correlation coefficient of 0.85 for the joint angles and 0.95 for the joint moments was achieved. The RMSE for the joint angle prediction was smaller than 4.8° and the nRMSE for the joint moment prediction was below 13.0%. Especially in the sagittal motion plane good results could be achieved. As the measured dataset is rather small, data was synthesized to complement the measured data. The enlargement of the dataset improved the prediction of the joint angles. While size did not affect the joint moment prediction, the addition of noise to the dataset resulted in an improved prediction accuracy. This indicates that research on appropriate augmentation techniques for biomechanical data is useful to further improve machine learning applications.

## 1. Introduction

Motion analysis, especially gait, in real-world environments gains more and more relevance in today's society. Since people are aging and want to retain their mobility, it is important to early detect abnormal gait patterns in order to prevent them from falling. To achieve this, the improvement of ambulatory motion analysis systems is relevant (Mundt et al., 2019a). Systems that are capable of determining motion kinematics and kinetics without expensive equipment and with less expert knowledge required will drastically increase the availability of motion analysis to a wider range of people. By providing wearable easy-to-use systems in daily life, risky motion patterns (e.g., in gait) might be identified before a major injury occurs or the onset of gait related diseases (Kobsar and Ferber, 2018; Majumder et al., 2019).

Gait is one of the main tasks of mobility. Baker et al. (2016) established four reasons for gait analysis: to diagnose a disease or injury, to assess the severity of a disease or injury, to monitor the progress of a disease or injury and to predict the outcome of an intervention. In all cases, long term or frequent monitoring of a person during daily life is desirable, thus allowing to identify any progression of a disease. To bring motion analysis into daily life, wearable sensors—especially inertial measurement units (IMUs)—have become increasingly popular (Caldas et al., 2017; Jarchi et al., 2018).

To extract joint angles from IMU data, the orientation of each sensor in a global reference system needs to be determined and a sensor-to-segment alignment performed. The most popular sensor fusion techniques for IMU-based motion analysis systems are (extended) Kalman filters or complementary filters (Gui et al., 2015). These filters fuse the signals of each single sensor of the IMU to determine its orientation. Either the data of the accelerometer and gyroscope only (Gui et al., 2015) or additionally the magnetometer data is used to identify the sensor orientation in a global reference system (Sabatini, 2006). The use of a magnetometer for the estimation of sensor orientation can be seen as a major limitation because magnetometers are highly susceptible to local disturbances in the magnetic field (de Vries et al., 2009; Teufl et al., 2018). Different attempts have been made either correcting magnetic disturbances or omitting the use of magnetometers at all (Ligorio and Sabatini, 2016; Teufl et al., 2018). However, another major issue of the commonly used approach is the (mal-)alignment of the sensor axes to physiological meaningful segment and rotation axes that define the anatomical model (Picerno, 2017; Robert-Lachaine et al., 2017; Mundt et al., 2019d). Several approaches have been suggested to overcome this problem: calibration postures or movements (Favre et al., 2009; Ferrari et al., 2010; Palermo et al., 2014), anatomical calibrations (Picerno et al., 2008; Bisi et al., 2015), post-trial calibration procedures (Hamacher et al., 2014; Li and Zhang, 2014) and more recently machine learning approaches (Zimmermann et al., 2018). While the use of calibration postures and movements will always be prone to errors because they are dependent on the execution of the subject (Seel et al., 2014; Picerno, 2017; Robert-Lachaine et al., 2017), the post-trial alignment prohibits fast data analysis. Therefore, the use of machine learning algorithms or the exploitation of kinematic constraints seems to be most promising. The most recent advancements of the kinematic-constraint-based approaches (Laidig et al., 2017, 2019; Müller et al., 2017; Nowka et al., 2019) have not been evaluated on gait analysis. Seel et al. (2014) evaluated the knee and ankle joint sagittal plane angle achieving deviations to the gold standard of less then 1°. Machine learning approaches have been undertaken by Findlow et al. (2008); Goulermas et al. (2008) achieving a mean correlation of about 0.70 for the sagittal plane joint angles. In recent work, we predicted joint angles based on simulated IMU data during gait achieving an accuracy higher than 0.86 (Mundt et al., 2019c).

Different approaches to determine the ground reaction force have been suggested and were systematically reviewed recently (Shahabpoor and Pavic, 2017). They concluded that the use of kinematic data as inputs reveals the highest practicality although showing lower accuracy than force plates. Additionally, the authors noted the limited validation of these methods for long-term measurements in real-life environment. This indicates that further research in this direction is useful, and if the aim is the evaluation of joint moments, a direct approach to determine these quantities might be advantageous. Different research has been undertaken in this direction, but less frequently. Ardestani et al. (2014) used a wavelet neural network to predict the 3D hip joint moments, the sagittal knee joint moment and the plantar flexion and eversion moment of the ankle joint during gait using GRF and EMG data as inputs. They reported normalized root-mean-squared errors of <20% and correlation coefficients ranging from 0.84 to 0.96. Johnson et al. (2018, 2019) used pre-trained convolutional neural networks for the prediction of the GRF and the knee joint moment during walking, running and sidestepping based on marker trajectories. They achieved a mean correlation higher than 0.85 for the knee joint moments and GRF. Analyzing normal gait, Hahn and O'Keefe (2008) estimated the sagittal plane lower limb joint moments based on demographic, anthropometric, kinematic, and EMG data. They achieved a coefficient of determination higher than 0.88, but they did not split their test set subject-wise, hence, data from subjects in the test set was also present in the training set. This leads to an improved accuracy (Saeb et al., 2017). Wouda et al. (2018) used inertial sensor data to determine the vertical GRF and the sagittal knee kinematics. For the joint angle prediction the correlation coefficient was larger than 0.83, and for the GRF larger than 0.90. In previous work, we used either marker trajectories or joint angles and the GRF as input data to predict all joint moments of the lower limbs during side stepping achieving a mean correlation higher than 0.86 (Mundt et al., 2019b). In a recent study, we used simulated IMU data to predict the joint moments during gait achieving a similar accuracy (Mundt et al., 2019c).

Despite the already good results, machine learning approaches have one important requirement: large datasets. These are - due to the novelty of the system - not openly available from IMU sensors. To overcome the lack of a large amount of data, their synthesizing is one reasonable solution (Young et al., 2011; Brunner et al., 2015; Zimmermann et al., 2018; Mundt et al., 2019c). Young et al. (2011) was the first who simulated IMU data from existing optical motion capture data to enlarge a dataset for pose estimation. This approach was taken a step further and validated by Brunner et al. (2015) and Zimmermann et al. (2018). In previous work, we simulated IMU data from optical data as well, but only validated the simulation based on a single participant (Mundt et al., 2019c). In this study, the validation of the simulation is continued and IMU data that was simulated based on optoelectronic data as well as measured IMU data is used as input parameters to train fully-connected feedforward neural networks. To be independent of a homogeneous magnetic field, the magnetometer data is not considered as input but the 3D angular rates and linear accelerations only. The major advantages of the proposed method are that the anatomical model is implicitly learned, hence no calibration postures or movements are necessary, and that joint kinematics and kinetics can be determined. We aim to predict the joint angles and joint moments of the lower limbs during gait and hypothesize that the use of combined simulated and measured data will achieve a higher accuracy than the use of measured data only. Furthermore, we hypothesize that the additional noise in measured data caused by soft tissue movements will decrease the prediction accuracy. We aim to provide a first step into the direction of in-field gait analysis based on IMUs and artificial intelligence.

## 2. Materials and Methods

**Figure 1:**
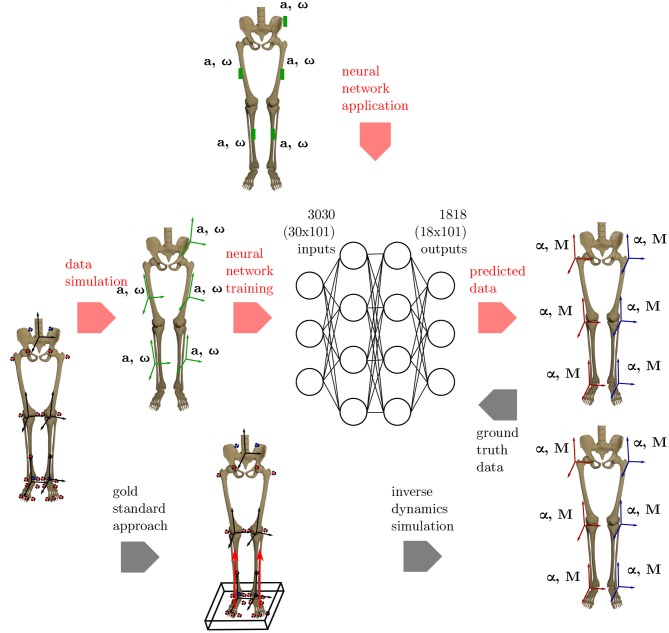
Overview of the methods applied. To get the ground truth information on the joint angles and joint moments of the lower limbs, the gold standard approach using an optical motion capture system and force plates to collect the data is used. Based on this data, inverse dynamics simulations are undertaken to calculate the joint angles and joint moments. Using the proposed ML method, inertial data (angular rate **ω** and acceleration **a**) is simulated from the optical data and used as inputs for an artificial neural network. Based on the ground truth joint angles and moments, the network learns the connection between the input and output data. The method is validated using an IMU system based on five sensors that are placed consistently with the simulated data.

An overview on the workflow of the proposed methodology is given in Figure 1.

### 2.1. Gold Standard Approach

All data used in this study was collected at the German Sport University Cologne. The studies were approved by the Ethical Committee of the German Sport University Cologne and all participants provided their informed written consent. The motion was recorded using an optoelectronic motion capture system (VICON^TM^, MX F40, Oxford, UK, 100–125 Hz) and two force plates (Kistler Instrumente AG, Winterthur, Switzerland, 1,000 Hz). In all studies, the participants were equipped with 28 reflecting markers that were attached to bony landmarks as depicted in detail in Mundt et al. (2019d) to create a rigid body model of the lower limbs. The marker trajectories and GRF were filtered using a zero-lag second-order low-pass Butterworth filter with a cut-off frequency of 6 Hz (Robertson et al., 2013) prior to calculating the joint angles and joint moments of the lower limbs with an anatomical landmark scaled model (Lund et al., 2015) using the AnyBody Modeling System^TM^ (Version 6.0, AnyBody Technology, Aalborg, Denmark). First, the kinematics are calculated using an overdetermined kinematic solver to optimize the markers using a least-squares approach. Afterwards, the models joint parameters are fitted to the subject-specific parameters before calculating the kinetics. All data was segmented into consistent sequences of 101 frames. For the kinematic data, full gait cycles were extracted based on an implementation of the foot contact algorithm proposed by Maiwald et al. (2009). For the joint moments a threshold-based segmentation of the stance phase was applied based on the force plate data. The joint moments were normalized to body height and weight of the participant.

### 2.2. Machine Learning Method

#### 2.2.1. Data Simulation

To derive the simulated IMU data, first, the anatomical coordinate systems of the biomechanical model need to be set up, because these coordinate systems are translated and rotated to match possible sensor positions before the derivatives are calculated to display the acceleration and angular rate.

**Figure 2:**
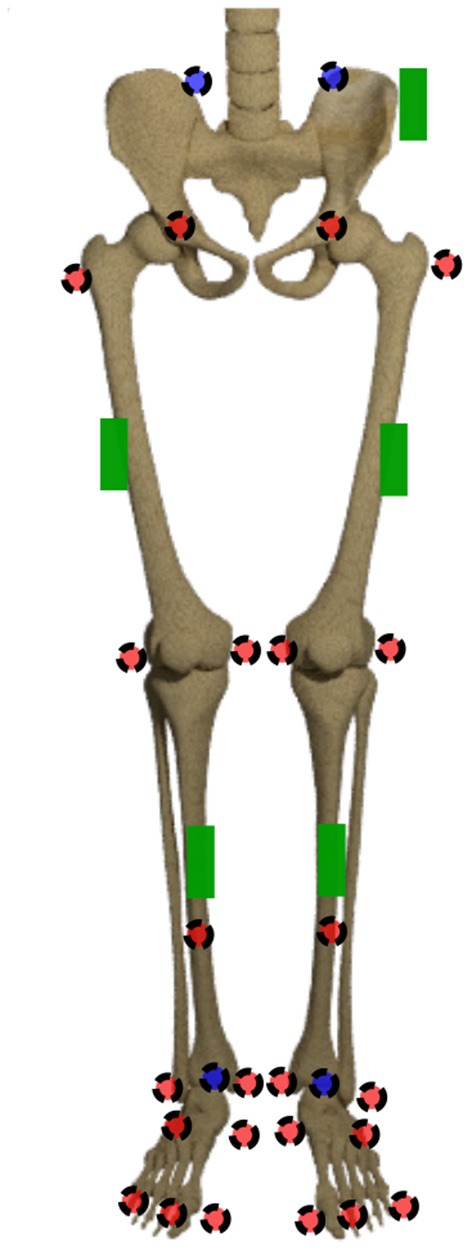
Marker set and sensor placement. The markers in the front are displayed in red, the ones at the back are displayed in blue. The green boxes display the IMU sensors.

The joint origins and segment coordinate systems of the hip, knee and ankle joint are calculated based on the marker trajectories. The marker set is displayed in Figure 2. The joint centers for pelvis and ankles are based on the recommendations of the International Society of Biomechanics (ISB) (Wu et al., 2002). The hip joint center is defined as per (Harrington et al., 2007). The definition of the knee joint center is based on Pennock and Clark (1990). After this step, five coordinate systems, one for the pelvis, two for the thighs and shanks, respectively, are set up. For ease of calculation, the coordinate systems are transformed to quaternions (Solà, 2017), denoted by **q**_*seg*_. For this purpose, the Hamilton convention is used:

(1) $$\begin{array}{l} i^{2}=-1,j^{2}=-1,k^{2}=-1\text{ and }ijk=-1 \end{array}$$

with *i, j*and*k* displaying the imaginary units of the quaternion. Any quaternion *Q* can thus be defined as:

(2) $$\begin{array}{l} Q=q_{0}+iq_{1}+jq_{2}+kq_{3}, \end{array}$$

with *q*_0_ being the scalar part of the quaternion and *iq*_1_+*jq*_2_+*kq*_3_ being the vector part. The quaternion can be interpreted as vector **q** in ℝ^4^, which is defined as:

(3) $$\begin{array}{l} \text{q}=\left(\begin{array}{c} \cos\left(\theta \right)\\ \text{u}\sin\left(\theta \right) \end{array}\right),\;∥\text{u}∥=1, \end{array}$$

where **u** = *u*_*x*_*i*+*u*_*y*_*j*+*u*_*z*_*k* is a unit vector describing the rotation axis and θ is a scalar describing the rotation angle.

In the following step, the anatomical coordinate systems are translated and rotated to match possible initial sensor positions and orientations. The rotation between the segment and sensor orientation can be described by **q**_ϕ_. The quaternion describing the orientation of the sensor in global space is calculated by:

(4) $$\begin{array}{l} {\text{q}}_{sensor}={\text{q}}_{seg}⊗{\text{q}}_{\phi }, \end{array}$$

with **q**_*sensor*_ describing the sensor orientation, **q**_*seg*_ describing the segment coordinate systems orientation in a global reference frame and **q**_ϕ_ describing the global quaternion rotation. The translation **x** of the segment coordinate system to the sensor position $\hat{\text{x}}$ is performed by:

(5) $$\begin{array}{l} \hat{\text{x}}={\text{x}}_{0}+\left({\text{q}}_{seg}⊗\text{x}⊗{\text{q}}_{seg}^{*}\right), \end{array}$$

with **x** and $\hat{\text{x}}$ being pure quaternions, with their components *x*_0_ and ${\hat{x}}_{0}=0$ yielding the sensor position in a global reference system and ${\text{q}}_{seg}^{*}$ denoting the conjugate of **q**_*seg*_. The original position, the joint center, is defined by **x**_0_.

In the following step, the angular velocity **ω** of each sensor can be calculated as the numerical quaternion derivative of the sensor orientation **q**_*sensor*_. For ease of readability, the subscript is omitted in the following. All quaternions **q** display the sensor's orientation. For two consecutive orientations **q**_*k*_ and **q**_*k*+1_, the local rotation Δ**q**_*l*_ of each sensor reads:

(6) $$\begin{array}{l} \Delta {\text{q}}_{l}={\text{q}}_{k}^{*}⊗{\text{q}}_{k+1}, \end{array}$$

which leads to:

(7) $$\begin{array}{l} \omega =\frac{2}{\Delta t}\frac{\Delta {\text{q}}_{lv}}{∥\Delta {\text{q}}_{lv}∥}\arctan\left(∥\Delta {\text{q}}_{lv}∥,\Delta q_{l0}\right). \end{array}$$

The subscripts *v* and 0 refer to the vector and the scalar part of the quaternion respectively. For further information on the derivation, see Solà (2017).

The linear acceleration of each sensor is calculated as the second derivative of the origin of the segment coordinate systems. This is achieved by reformulating a Taylor series expansion around **x**_*k*_ with *k* = 2, …, *n* − 1 with *n* being the last time step. For **x**_1_ and **x**_*n*_ the one-sided forward and backward differences need to be used respectively. The approximation can be improved for **x**_2_ to **x**_*n*−1_ by applying the central differences scheme (Atkinson and Han, 2005). In summary, this yields the following equations for the velocities:

(8) $$\begin{array}{l} {\text{v}}_{1}=\frac{{\text{x}}_{2}-{\text{x}}_{1}}{\Delta t},\;{\text{v}}_{k}=\frac{{\text{x}}_{k+1}-{\text{x}}_{k-1}}{2\Delta t},\;{\text{v}}_{n}=\frac{{\text{x}}_{n}-{\text{x}}_{n-1}}{\Delta t}. \end{array}$$

The same procedure can be applied again to derive the second order differentiation for **a**_*k*_. Analogously to **v**_*k*_, the same restrictions hold for the first and last time steps. Thus, the finite difference approximations of the accelerations are:

(9) $$\begin{array}{r} {\text{a}}_{1}=\frac{{\text{x}}_{3}-2{\text{x}}_{2}+{\text{x}}_{1}}{\Delta t^{2}},\;{\text{a}}_{k}=\frac{{\text{x}}_{k+1}-2{\text{x}}_{k}+{\text{x}}_{k-1}}{\Delta t^{2}},\\ {\text{a}}_{n}=\frac{{\text{x}}_{n}-2{\text{x}}_{n-1}-{\text{x}}_{n-2}}{\Delta t^{2}}. \end{array}$$

To transform the numerical derivatives into the actual sensor readings the different signs of gravity and motion need to be considered to define the acceleration of the sensor in the global reference system **a**_*g*_:

(10) $$\begin{array}{l} {\text{a}}_{g}=-\text{a}+\text{g}. \end{array}$$

The different signs are caused by the working principle of accelerometers that are used in inertial measurement units. Accelerometers are based on the inertial force of a small mass acting upon a piezoelectric element (Elwenspoek and Wiegerink, 2001). Thus, the gravitational acceleration directly translates to the sensor reading while the acceleration of the sensor origin results in an inertial force in the opposite direction of the segment acceleration. This means that the sign of a needs to be inverted while the sign of **g** remains unchanged. To describe the sensor readings in its local coordinate system the following transformation is necessary:

(11) $$\begin{array}{l} {\text{a}}_{l}={\text{q}}_{sensor}^{*}⊗{\text{a}}_{g}⊗{\text{q}}_{sensor}, \end{array}$$

with **a**_*l*_ displaying the linear acceleration of a sensor. The acceleration **a**_*g*_ and **a**_*l*_ are pure quaternions, with their components *a*_*g*0_ and *a*_*l*0_ = 0.

As the sensor is assumed to be a rigid body, its local position and orientation can be exactly described by six degrees of freedom, three translations and three rotations, described by the translation vector **x** (see Equation 5) and the rotation vector **q**_ϕ_ (see Equation 4). In order to optimize these quantities, a vector **z** = [*x*_1_, *x*_2_, *x*_3_, *q*_ϕ1_, *q*_ϕ2_, *q*_ϕ3_] is defined. We fit the values using the following objective function:

(12) $$\begin{array}{l} \Theta \left(\text{z}\right)=\overset{N_{t}}{\underset{n_{t}=1}{\sum }}{\left(\omega_{n_{t}}^{\left(m\right)}-\omega_{n_{t}}^{\left(s\right)}\left(\text{z}\right)\right)}^{T}\left(\omega_{n_{t}}^{\left(m\right)}-\omega_{n_{t}}^{\left(s\right)}\left(\text{z}\right)\right)\\ \;+\overset{N_{t}}{\underset{n_{t}=1}{\sum }}{\left({\text{a}}_{n_{t}}^{\left(m\right)}-{\text{a}}_{n_{t}}^{\left(s\right)}\left(\text{z}\right)\right)}^{T}\left({\text{a}}_{n_{t}}^{\left(m\right)}-{\text{a}}_{n_{t}}^{\left(s\right)}\left(\text{z}\right)\right), \end{array}$$

subject to,

(13) $$\begin{array}{l} x_{min}\leq x_{i}\leq x_{max},\;i=1,2,3 \end{array}$$

(14) $$\begin{array}{l} 0\leq \phi_{i}\leq \frac{\pi }{2},\;i=1,2,3. \end{array}$$

In this formulation, $\omega_{n_{t}}^{\left(m\right)}$ and $\omega_{n_{t}}^{\left(s\right)}\left(\text{z}\right)$ denote the angular rates in the three dimensional space. The superscripts (m) and (s) describe the measured and simulated values. Equivalently, ${\text{a}}_{n_{t}}^{\left(m\right)}$ and ${\text{a}}_{n_{t}}^{\left(s\right)}\left(\text{z}\right)$ denote the acceleration in three dimensions. We additionally defined the minimum and maximum allowed deviation of the positions *x*_*min*_ and *x*_*max*_ to be ±50 mm as well as the maximum allowed orientation deviation $\frac{\pi }{2}$. The constrained optimization problem was solved using the interior-point algorithm (Byrd et al., 1999), which is implemented in MATLAB. The defined constraints do not allow for an arbitrary sensor positioning but for a compensation for positioning and orientation errors in the range specified by the constraints. For this purpose, the sensor-to-segment-assignment needs to be consistent.

#### 2.2.2. Neural Network Implementation

The python library Tensorflow (Abadi et al., 2015) was used to implement a fully-connected feedforward neural network (Koeppe et al., 2019). Artificial neural networks work as universal function approximators. Instead of explicitly programming the solution of one specific task, they learn from existing (big) data. Artificial neural networks have been inspired by the structure of the human brain, consisting of single neurons that add up to layers to increase the capacity of the network. Using multiple (hidden) layers with a specified number of neurons, the capacity of the network can be adapted (Mundt et al., 2019c). Fully-connected neural networks need time-normalized data as inputs, hence, only an offline analysis can be performed.

Different networks were trained for the prediction of joint kinematics and the joint kinetics based on different datasets. The first one is a collection of optical motion capture data of gait trials previously collected at the German Sport University Cologne. The dataset comprised 93 participants (38 female, 39.9 (18–75) years, 72.6 (47.1–97.6) kg, 1.73 (1.54–1.98) m, BMI 24.3 (17.5–31.6) kg m^−2^). A number of 24 participants underwent knee arthroplasty 1.8 ± 0.4 years post-surgery prior to gait analysis (Komnik et al., 2015). The optical data collected in this study was additionally added to the dataset as well.

After validation of the simulated data, the neural network was trained using the accelerations and angular rates of the five sensors depicted in Figure 2 as input data, which resulted in 30 inputs. One sensor was placed at the pelvis, one on each thigh and one on each shank. The sensors were not aligned to the segments, because the dataset is supposed to cover the complete range of orientations and positions due to the data simulation. Thereby, the neural network can learn to handle the differences. A kinematic model was trained to predict the 18 joint angles of the lower limbs, while a kinetic model was trained to predict the 18 joint moments of the lower limbs. Because we use a fully-connected feedforward neural network, no time-dependencies can be covered by the neural network (Goodfellow et al., 2016). Therefore, all data was time normalized and unrolled before being input to the network. This resulted in an input layer of size 30 × 101 = 3,030 and an output layer of 18 × 101 = 1,818. For the analysis, only the nine angles/moments of the foot touching the ground are evaluated.

**Figure 3:**
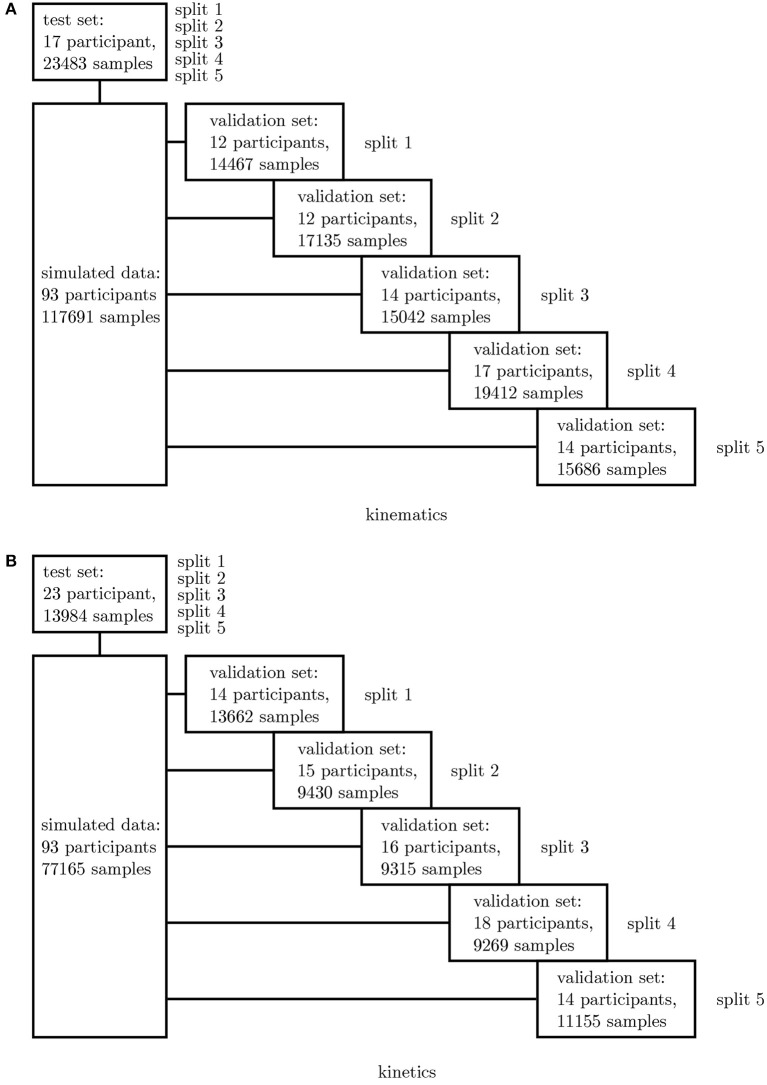
Overview of the 5-fold cross-validation process. The dataset for the kinematics **(A)** and kinetics **(B)** differ and were treated separately.

In a first step, all simulated IMU data was used to determine the best network architecture and hyperparameters for the application using a 5-fold cross-validation. Therefore, one fixed test set was split from the complete dataset as well as five different validation sets. The split was undertaken randomly ensuring that no overlapping between the sets occurred (cf. Figure 3). A grid search was conducted to optimize the architectures and hyperparameters.

### 2.3. Validation

#### 2.3.1. Experimental Set-Up

Thirty healthy subjects (12 female, 28.1 ± 6.0 years, 72.3 ± 12.7 kg, 1.77 ± 0.07 m) participated in this study that was approved by the Ethical Committee of the German Sport University Cologne. All participants provided their informed written consent. Each subject performed 10 level walking trials at five different speeds: 0.8 m s^−1^, 1.1 m s^−1^, 1.4 m s^−1^, 1.7 m s^−1^ and 2.0 m s^−1^ ±10% on a 5 m walkway. According to the set-up of all previous experimental investigations, each participant was equipped with 28 retro-reflective markers to capture the motion by 12 infrared cameras (125 Hz, VICON^TM^, MX F40, Oxford, UK). Simultaneously, the participants were equipped with five sensors of a custom low cost IMU system (100 Hz, TinyCircuits, Akron, OH, USA) with an associated microcontroller (Atmel ATmega328P) and a WIFI-board (Atmel ATWINC1500). An Android application was developed to collect the data on a smartphone (Mundt et al., 2018b). The marker set and sensor placement are displayed in Figure 2. The sensors were only roughly aligned to the segments but a consistent sensor-to-segment assignment was used. The data of seven subjects was excluded from this study due to connectivity issues, hence, data of 23 participants is presented.

#### 2.3.2. Data Synchronization

The synchronization of the IMU system and the optoelectronic system cannot be performed automatically. Therefore, a synchronization algorithm was developed. For this purpose, the simulated medio-lateral acceleration of the pelvis was used. An average position and orientation estimation of the pelvis sensor was chosen. For the actual synchronization an optimization problem was defined. We obtained the minimization problem with the following mean-square objective function Υ:

(15) $$\begin{array}{l} \Upsilon \left(\delta \right)=\overset{N_{t}}{\underset{n_{t}=1}{\sum }}{\left(a_{n_{t}}^{\left(m\right)}-a_{n_{t}}^{\left(s\right)}\right)}^{2}. \end{array}$$

**Figure 4:**
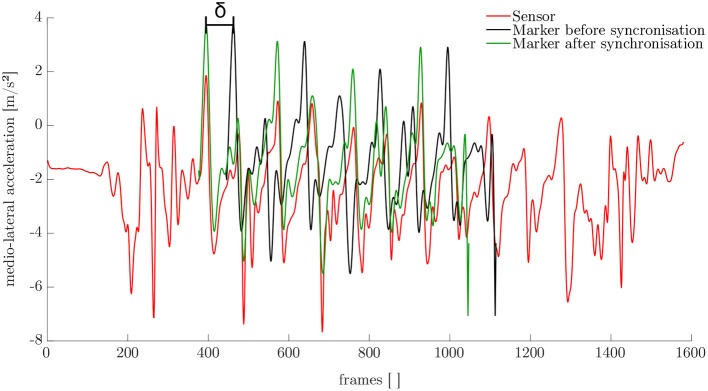
Results of the synchronization based on the medio-lateral acceleration of the pelvis.

Here, $a_{n_{t}}^{\left(m\right)}$ and $a_{n_{t}}^{\left(s\right)}$ denote the measured and simulated acceleration of the pelvis in the medio-lateral direction. The value δ is the distance between the first local maximum peak in the measured and simulated data (cf. Figure 4). The start value for the optimization was chosen based on the output of the optical motion tracking system. The optimization problem is iteratively solved using the Nelder-Mead Simplex method (Lagarias et al., 1998) already implemented in MATLAB. After synchronization, the optical motion capture data and the inertial sensor data was segmented into steps as described in section 2.1.

#### 2.3.3. Simulation Framework

First, the simulation framework was validated. For this purpose the optimum position and orientation of each sensor were determined for each trial. Hence the sensors were fixed once for each subject during the complete experiment, the best estimation was determined for each subject based on the root-mean-squared error. Afterwards, all data was simulated again based on the optimized values. Thereby, a valid solution representing the differences in placement during the experiments was found. This procedure resulted in 23 (one per subject) optimized initial values. This information was used to generate a large simulated dataset based on the optical data of the former studies. All sensor positions and orientations found during the experiments were covered.

#### 2.3.4. Neural Network Application

**Figure 5:**
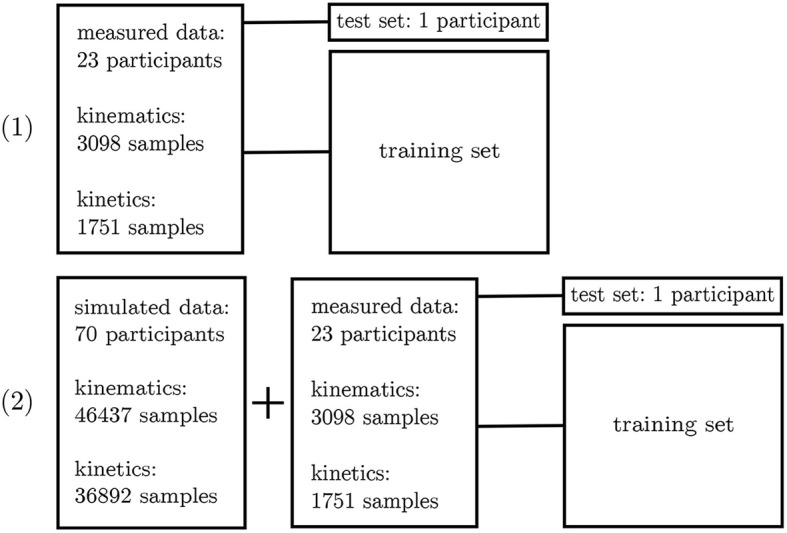
Overview of the leave-one-out validation process. Kinematics and kinetics were treated separately.

The inertial sensor data was used to validate the simulation framework presented. Afterwards, the neural network application was verified on the measured data. For this purpose, a leave-one-out cross-validation (Arlot and Celisse, 2010) was performed to enable the performance analysis of the neural network on single subjects. Two different scenarios were investigated: (1) all experimental data—besides one subject—was used for training purposes and (2) all experimental data—besides one subject—plus the simulated data was used for training. Since the best architecture and hyperparameters have been found in the first step, no further validation set is necessary. The left-out subject served as test set (cf. Figure 5).

### 2.4. Data Analysis

First, the results of the data simulation are presented. Afterwards, the results of the 5-fold cross-validation and the grid search to find the optimum neural network parameters are displayed. Finally, the results of the leave-one-out cross-validation based on measured data only and on the combined—measured and simulated—data are presented. To evaluate the simulation and prediction accuracy, the correlation coefficient was calculated. Furthermore, the RMSE was determined for the joint angles and the nRMSE (normalized RMSE to the range of the data) for the joint moments. A paired *t*-test and the effect size were calculated on the RMSE/nRMSE values. Additionally, the maximum joint angles and joint moments were calculated to evaluate the performance on this scalar parameter. An ANOVA and *post-hoc t*-test with Bonferroni correction as well as the effect size were calculated on the prediction of the maximum joint angles and joint moments. For each subject one mean step was considered.

## 3. Results

### 3.1. Neural Network Parameters

The best parameters for the neural network were evaluated based on the simulated dataset. An initial learning rate α = 10^−4^ and an increasing batch size of 16-32-32-64 samples during the four phased training schedule performed best for both the kinematic and kinetic model. For the kinematic model, two hidden layers with 4,000 and 6,000 neurones, a dropout rate of 20% and 12,500 training steps per phase revealed the highest accuracy. For the kinematic model, two hidden layers with 6,000 and 4,000 neurones, a dropout rate of 40% and a number of 15,000 training steps per phase showed the best results.

### 3.2. Data Simulation

**Figure 6:**
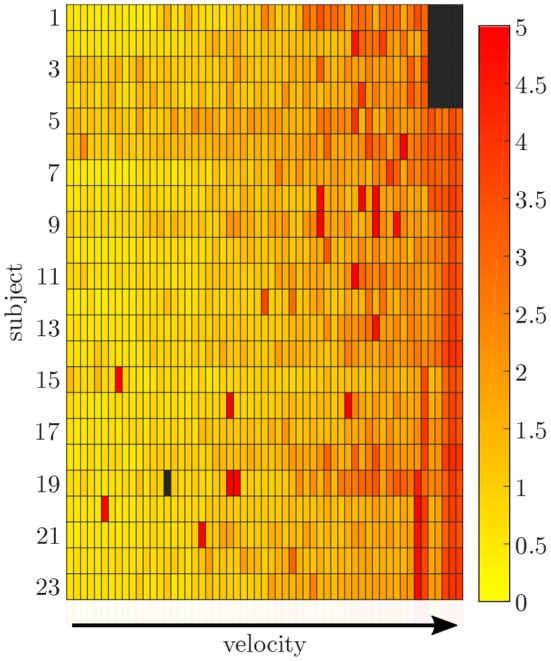
Root-mean-squared error between the measured and simulated data exemplarily displayed for the pelvis sensor. With an increasing gait velocity the simulation error increases. Some trials show outliers with larger errors during slow walking.

The simulation of the data for all sensors was based on one fixed sensor position and orientation for each subject. The mean RMSE between the measured and simulated data is displayed in Figure 6. With an increase in gait velocity, the RMSE increased while the correlation coefficient decreased. The simulated data of the pelvis sensor achieved the highest accuracy (r_pelvis_ = 0.95 ± 0.08), while the accuracy for the sensors of the legs is slightly lower (r_rightthigh_ = 0.88 ± 0.12, r_leftthigh_ = 0.91 ± 0.08, r_rightshank_ = 0.91 ± 0.11, r_leftshank_ = 0.92 ± 0.10).

### 3.3. Five-Fold Cross-Validation

**Figure 7:**
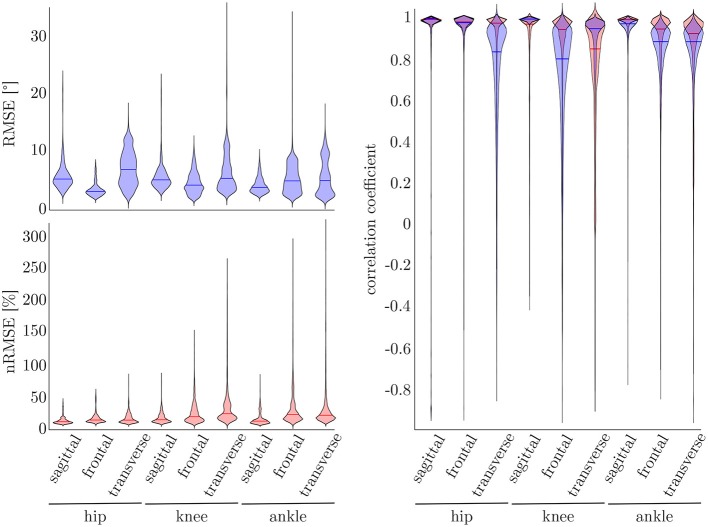
On the right, the distribution of the correlation coefficient for the kinematic (blue) and kinetic (red) model is displayed. Additionally, on top, the distribution of the RMSE for the kinematic and on the bottom the distribution of the nRMSE for the kinetic model can be found. The violin's width displays how much data is accumulated, while the height shows the range of the distribution. The horizontal line indicates the median value of the distribution.

To find the best model architecture and optimize the hyperparameters, a 5-fold cross-validation was undertaken using the simulated data of all subjects. The results for the kinematic and kinetic model are displayed in Figure 7. For both models the mean correlation coefficient was very similar on new test data (kinetics: 0.86, kinematics: 0.87). The prediction of the knee joint frontal plane angle and the transverse moment showed the weakest correlations, while the prediction of the joint moments showed the highest accuracy in all planes for the hip joint (>0.91) and the joint angle prediction in all joints in the sagittal plane (>0.95). Additionally, the kinetic predictions showed less outliers than the kinematic predictions. Over all, the RMSE of the joint angle prediction was smaller than 6.0° for all joints and motion planes with a mean value of 4.1° and the nRMSE of the joint moment prediction was smaller than 25.5% with a mean value of 15.5%.

### 3.4. Leave-One-Out Cross-Validation

**Figure 8:**
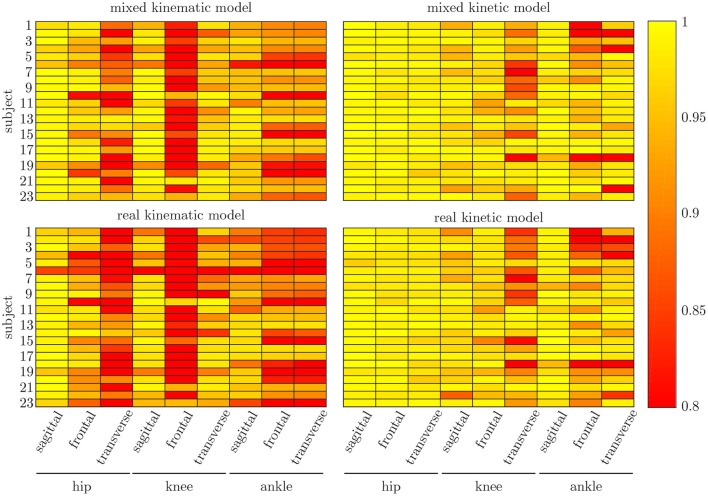
Mean correlation coefficient for each joint and motion plane of each subject in the test set. On top, the results for the combined input data are displayed, while on bottom, the results of the model using measured data only are depicted. There are only small differences between both models, while there are distinct differences in the different motion planes and between subjects.

**Figure 9:**
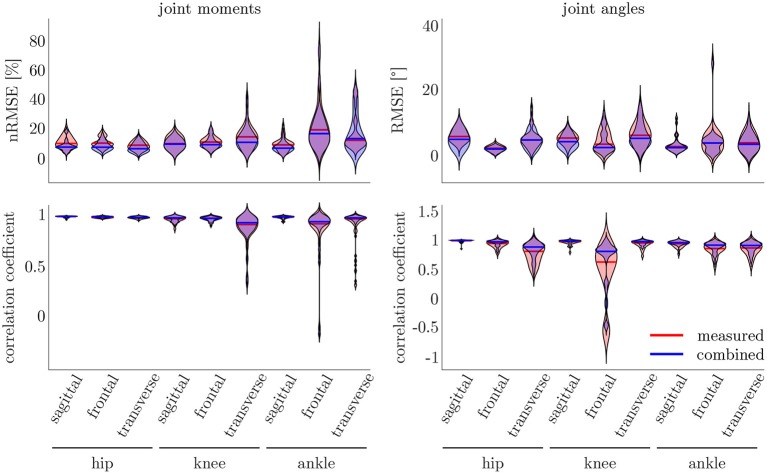
On the right, the distribution of the RMSE and the correlation coefficient for the kinematic data is displayed. On the left, the distribution of the nRMSE and the correlation coefficient for the kinetic model can be found. The results for the measured data as inputs is displayed in red, while the results for the combined data inputs are displayed in blue. The violin's width displays how much data is accumulated, while the height shows the range of the distribution. The horizontal line indicates the median value of the distribution.

The leave-one-out cross-validation shows the performance of the model for each subject when trained on all other subjects' measured and simulated data. As the hyperparameters were fixed from the 5-fold cross-validation, no further validation set was necessary. There were only small differences in the correlation of the predicted and measured data using measured data only or the combined data set (cf. Figure 8). This finding was supported by the results of the *t*-test: there were differences between the two kinematic models in the prediction of all sagittal joint angles and the frontal hip joint angles (r_hipsagittal_<0.001, r_hipfrontal_<0.001, r_kneesagittal_<0.001, r_anklesagittal_=0.043). For the kinetic model, differences in all hip joint moments and the ankle joint sagittal moment were found (r_hipsagittal_<0.001, r_hipfrontal_<0.001, r_hiptransverse_<0.001, r_anklesagittal_=0.006). Additionally, the correlation coefficient showed distinct differences between the motion planes: the prediction accuracy of the hip joint angle in the transverse plane, the knee joint angle in the frontal plane and the ankle joint angle in the frontal and transverse plane was lower than in the other planes. The prediction of the joint moments was more accurate although there were some subjects showing lower correlation coefficients in some features. The same behavior could be found when analyzing the distribution of the results (cf. Figure 9). Those parameters with weaker correlations showed a wider spread and more outliers in the distribution of the RMSE/nRMSE and correlation coefficient. The mean correlation of the models was r_kinematicmeasured_=0.85, r_kinematiccombined_=0.89, r_kineticmeasured_=0.95 and r_kineticcombined_=0.95. The mean error was RMSE_kinematicmeasured_=4.8°, RMSE_kinematiccombined_=4.3°, nRMSE_kineticmeasured_=13.0% and nRMSE_kineticcombined_=11.6%.

Compared to the model used for the 5-fold cross-validation that was based on simulated data only, the accuracy was similar for the kinematic model and even higher for the kinetic model. With regard to Figures 7, 9, the mean accuracy was similar for the cross-validation and the leave-one-out validation, but the number of outliers was decreased.

**Figure 10:**
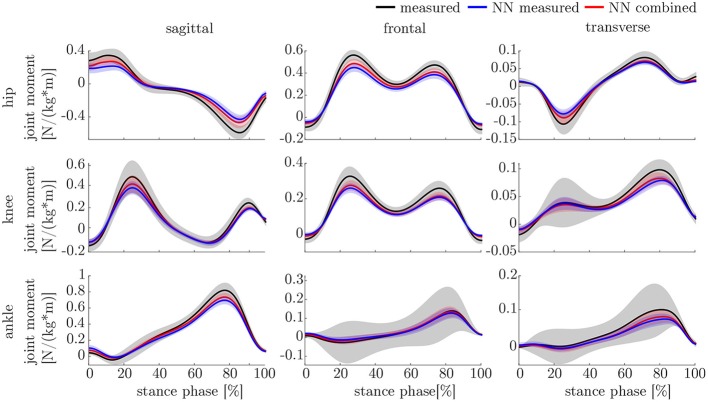
Overview of the mean and standard deviation of the joint moments of the 23 subjects.

**Figure 11:**
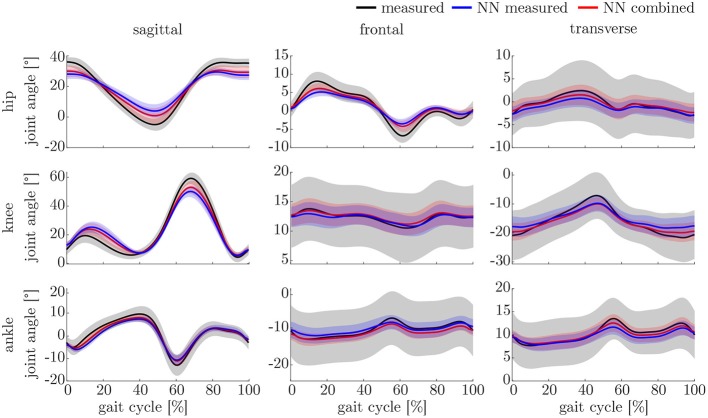
Overview of the mean and standard deviation of the joint angles of the 23 subjects.

The ANOVA showed significant differences in the maxima between the predicted and measured joint angles and moments. The *post-hoc t*-test indicated significant differences between both the measured and predicted and the two predicted values. The prediction of the peak joint moments showed more significant differences than the prediction of the peak joint angles (cf. Table 1). The trends of the statistical analysis can also be seen in Figures 10, 11.

**Table 1:** Results of the statistical analysis of the peak prediction.

	**Joint angles**					
	**Measured vs. real**		**Measured vs. combined**		**Real vs. combined**	
	***p***	**d**	***p***	**d**	***p***	**d**
Hip sagittal	<0.001^⋆^	1.867	<0.001^⋆^	1.392	0.004^⋆^	0.676
Hip frontal	<0.001^⋆^	1.293	<0.001^⋆^	0.948	<0.001^⋆^	0.924
Hip transverse	0.085	0.377	0.226	0.260	0.080	0.383
Knee sagittal	<0.001^⋆^	2.580	<0.001^⋆^	1.683	<0.001^⋆^	1.103
Knee frontal	0.324	0.210	0.566	0.121	0.061	0.412
Knee transverse	0.123	0.335	0.104	0.354	0.724	0.074
Ankle sagittal	0.005^⋆^	0.645	0.018	0.536	0.013^⋆^	0.561
Ankle frontal	0.412	0.174	0.211	0.269	0.141	0.319
Ankle transverse	0.035^⋆^	0.470	0.174	0.293	0.006^⋆^	0.638
	**Joint moments**					
	**Measured vs. real**		**Measured vs. combined**		**Real vs. combined**	
	***p***	**d**	***p***	**d**	***p***	**d**
Hip sagittal	<0.001^⋆^	1.659	<0.001^⋆^	1.033	<0.001^⋆^	1.452
Hip frontal	<0.001^⋆^	2.283	<0.001^⋆^	1.850	<0.001^⋆^	1.036
Hip transverse	0.004^⋆^	0.674	0.010^⋆^	0.592	0.021	0.517
Knee sagittal	<0.001^⋆^	0.854	0.004^⋆^	0.671	0.011^⋆^	0.576
Knee frontal	<0.001^⋆^	1.193	<0.001^⋆^	0.961	0.003^⋆^	0.690
Knee transverse	<0.001^⋆^	0.977	0.002^⋆^	0.735	0.031	0.482
Ankle sagittal	<0.001^⋆^	1.296	<0.001^⋆^	0.901	0.001^⋆^	0.839
Ankle frontal	0.041	0.452	0.108	0.350	0.078	0.386
Ankle transverse	0.038	0.460	0.079	0.384	0.087	0.373

*Significant results are indicated by ^⋆^*.

## 4. Discussion

### 4.1. Data Processing

The data processing was one major challenge in this study because there was no possibility to synchronize both measurement systems automatically. The developed approach based on optimization might lead to errors, especially because gait is a cyclic motion. Sequences were not filtered for outliers, which might also cause outliers in the prediction. We decided not to remove outliers from the dataset to minimize the pre-processing on the data and observe the networks' capability to handle this data. The simulated data can represent the measured IMU data well, showing good correlations when using a fixed sensor position and orientation for the calculation of angular rate and linear acceleration for each subject. Higher gait velocities cause larger deviations between the measured and simulated data (cf. Figure 6), which can be attributed to soft tissue movements that cause noise in the measured data that is not included in the simulated data. The optical markers placed on bony landmarks are the basis for the simulated IMU data, while the physical IMU sensors are placed on the body as displayed in Figure 2. Hence, the markers and sensors experience different soft tissue movements that correlate with the gait velocity and increase the error. However, the mean correlation coefficients indicate an overall good accuracy. The correlation found by Young et al. (2011); Brunner et al. (2015) and Zimmermann et al. (2018), who proposed frameworks for simulating IMU data, is comparable to the results presented in this study. Young et al. (2011) and Brunner et al. (2015) tested their simulator for leg swinging and single rigid body movements achieving very good correlation coefficients (*r* >0.97). Since during this motion no impact occurs which causes soft tissue movements the results are better than the ones achieved in this study. Zimmermann et al. (2018) evaluated their simulation approach on a pure rigid body motion (*r* >0.97) and during gait (*r*_*acc*_ >0.57 and *r*_*gyr*_ >0.93). These results support the explanation that the impact causes soft tissue movements during gait and limits the comparability between simulated and measured data. However, the results of this study are slightly better than in the study of Zimmermann et al. (2018).

### 4.2. Cross-Validation

The kind of validation strategy chosen can highly influence the results (Little et al., 2017; Saeb et al., 2017). We aimed to find the best model parameters and hyperparameters using a 5-fold cross-validation based on the simulated dataset only. Thereby, it was possible to exclude a fixed test set of a representative size as well as a randomly chosen validation set that covers most gait patterns. We ensured, that no data of any subject was part of more than one subset to avoid bias (Saeb et al., 2017). Simultaneously, it was possible to undertake a grid search on the best parameters and hyperparameters in a reasonable time frame. Afterwards, we performed a leave-one-out cross-validation on the new data collected in this study. This led to 23 training runs per model. Thereby we aimed to analyze the prediction accuracy on single subjects. Using this two-stage validation approach, it is possible to use as much data as possible for training the models, because there is no validation set necessary as the hyperparameters were fixed. As a side effect of the 5-fold cross-validation, it is possible to additionally compare the results of measured and combined input data to only simulated input data. However, this approach might also cause a suboptimal accuracy on the measured data, because the network architecture and hyperparameters were tuned to optimize the prediction on the simulated dataset only, which is larger than the measured dataset and the combined one.

### 4.3. Kinematics and Kinetics

The lower accuracy of the prediction of the kinematics indicates that it is a more difficult task for the neural network to predict the joint angles than the joint moments. This might be attributed to the closer physical relationship of acceleration and (normalized) joint moments. Additionally, the joint angles do not start at a value around zero, which leads to a more difficult initial value problem than for the joint moments. Therefore, the prediction of the kinematics profits from an enlarged dataset, which can be seen in the increased prediction accuracy from measured over combined to simulated data. In contrast, the kinetics prediction seems to improve with additional noise in the input data instead of the larger dataset. This can be seen in the increased prediction accuracy in the combined and measured dataset compared to the simulated data only, which does not include the larger soft tissue movements the sensors experience in faster walking. Soft tissue movements also affect the calculation of joint angles and joint moments, which is a limitation in every motion analysis. One disadvantage of the simulated IMU data is that it does not include the same soft tissue movements as the marker trajectories.

Both, the kinematic and the kinetic model, are not able to cover the complete variance of the measured data (cf. Figures 10, 11). This might be improved by further increasing the dataset and the noise of the inputs. Therefore, research on data augmentation should be further emphasized. The higher variance in the results of the cross-validation models compared to the leave-one-out model might be attributed to the dataset. The dataset used for the cross-validation includes participants with larger demographic differences as well as knee arthroplasty patients while the leave-one-out dataset comprises young participants without any impairment only. For the cross-validation, one test set was split from the complete dataset, while for the leave-one-out validation only the participants of this study served as test set (cf. Figures 4, 5). Additionally, Figure 7 displays each single trial while in Figure 9 the mean results of each participant are displayed.

Comparing the results of this study to the literature is difficult, because this is—to the authors knowledge—the first time that IMU data was used to predict the joint angles and moments in all three motion planes. Especially the analysis of kinematic parameters using machine learning is not well investigated so far. As displayed in Figure 8, the correlation coefficient is larger than 0.8 in the sagittal plane for all subjects regarding the joint angles and even higher in all motion planes regarding the joint moments. Only Findlow et al. (2008) used an approach based on neural networks to predict joint angles. We achieved a higher accuracy in our study, which is probably caused by the larger dataset, more sensors involved and an improved computing power and algorithms compared to their study undertaken in 2008. Another approach is the use of kinematic constraints to determine joint angles from IMU data. Based on different joints, this approach reveals very good results (Müller et al., 2017; Laidig et al., 2019; Nowka et al., 2019). Nevertheless, it was not analyzed recently for gait analysis. Seel et al. (2014) achieved already good results when analyzing the sagittal knee and ankle joint angles with an mean RMSE of 3.3° and 1.6°, respectively. These results are slightly better than our results with 4.62° and 2.42°. It might be possible to improve the accuracy of the proposed method when also specializing on single joint angles or adding additional sensors to the model. In a previous study, we could achieve an error smaller than 2.5° in all joints and motion planes, when using simulated data only and additional data for the feet sensors (Mundt et al., 2019c). Zihajehzadeh and Park (2017) used a more common approach for the joint angle estimation based on an adapted Kalman Filter that does not use magnetometer data for the orientation estimation. They achieved RMSE values smaller than 3.5° for the three sagittal plane angles and the hip adduction/abduction during walking. Teufl et al. (2018) also investigated the use of magnetometer free joint angle estimation. Their method achieved mean RMSE values of <2.3° for all joints and motion planes. This results is very promising, although it needs to be considered that the biomechanical model was set up using optical motion capture data. In a previous study, we could show that the differences in joint angle estimation is mainly based on the definition of the rotation axes used by the IMU systems (Mundt et al., 2019d), what we aimed to overcome with the neural network approach that implicitly learns the biomechanical model during the training process.

There is more research on estimating joint kinetics, but none was undertaken using IMU sensors as input data to predict all 3D lower limb joint moments. In one of our previous studies, we used joint angles as input parameters to predict joint moments (Mundt et al., 2018a). We achieved slightly better results than in this study. In another study using simulated IMU data, the joint moment prediction resulted in an nRMSE of 12.16%, which is slightly lower than in this study although using additional sensors on the feet (Mundt et al., 2019c). This further supports the hypothesis that more noise in the data is favorable for the joint moment prediction and that it might be useful to investigate the relevant features for the neural network. For this purpose, Horst et al. (2019) suggested to use the Layer-Wise Relevance Propagation technique. Another approach might be the use of principle component analysis to analyze the sensitivity of inputs and outputs (Ardestani et al., 2015).

In future work, it might be useful to investigate a two-staged approach: first, predict the joint angles from IMU data and second, use the estimated joint angles to predict the joint moments. However, for this approach the joint angle estimation needs to be further improved. It might also be conceivable to take this approach the other way round, using joint moments as input data to predict joint angles, because the joint moments show better results so far. It might also be feasible to add (estimated) joint angles or joint moments to the IMU input data for further improvement. Additionally, the choice of another kind of artificial neural network, e.g., long short-term memory (LSTM) or convolutional neural networks (CNN), might be suitable for the underlying task. Especially due to the high number of inputs (30 features times 101 time frames) these neural networks might outperform the fully-connected feedforward neural network, that was used in this study. While a fully-connected feedforward neural network uses flattened data (no time dependency) as inputs, LSTMs and CNNs preserve the time dependency. Thereby, it might be easier for these networks to extract the most relevant features from the data (Goodfellow et al., 2016). In this study, we analyzed short sequences of motion only. During these sequences, no gyroscope drift could be observed. For future research, to bring this method further toward application, this aspect needs to be considered. Another sensor system might overcome this limitation. We also only analyzed straight walking. Most probably, this method can also be applied to more diverging motion, when this motion is present in the training dataset. It might even lead to an improved accuracy, when using a dataset showing more variance (Mundt et al., 2019b). Further analysis on the relevant features for the neural network to predict the joint angles and moments will be valuable to maybe reduce the number of sensors necessary for the prediction and thereby decreasing the complexity of the model. Further validation of the method with a larger amount of measured data should be undertaken.

## 5. Conclusion

This study analyzed the ability of a fully-connected feedforward neural network to predict joint angles and joint moments of the lower limbs based on IMU data. Our hypothesis, that simulated data can support the learning of the neural network can be accepted for the joint angle prediction while it can only be partly accepted for the prediction of joint moments. Our second hypothesis, that noise in the input data decreases the prediction accuracy can be rejected. For the kinetic prediction, the noise attributed to soft tissue movements improves the prediction accuracy and seems to be more important than the size of the dataset. The prediction of the joint angles is not affected by noise. Therefore, it needs to be evaluated if the prediction can be further improved using a simulated dataset containing soft tissue movement induced noise in the input data. Thereby, the measured data might be better represented and the learning of the neural network improved. Nevertheless, the results already demonstrate the high potential of the approach and support further research on neural networks in gait analysis. Besides the aforementioned data augmentation, different kind of neural networks (e.g., recurrent or convolutional neural networks) should be investigated on the task in future work. Thereby, data that is not time normalized could be used, hence the gait velocity could be included in the data. For the analysis of clinically relevant parameters, it might also be suitable to train patient-specific models to achieve a higher accuracy (Saeb et al., 2017).

## Data Availability Statement

The datasets generated for this study are available on request to the corresponding author.

## Ethics Statement

The studies involving human participants were reviewed and approved by Ethikkommission der Deutschen Sporthochschule Köln. The patients/participants provided their written informed consent to participate in this study.

## Author Contributions

MM and SD carried out the experiment. MM, AK, and TW undertook the data analysis. MM and FB wrote the manuscript. WP and BM supervised the multidisciplinary project and revised the manuscript.

### Conflict of Interest

The authors declare that the research was conducted in the absence of any commercial or financial relationships that could be construed as a potential conflict of interest.
